# Flexible and rigid ureteroscopy in outpatient surgery

**DOI:** 10.1186/s12894-016-0124-z

**Published:** 2016-01-28

**Authors:** Abeni Oitchayomi, Arnaud Doerfler, Sophie Le Gal, Charles Chawhan, Xavier Tillou

**Affiliations:** Urology and Transplantation Department, University Hospital of Caen, CHU Cote de Nacre, Avenue de Cote de Nacre, 14033 Caen, France

**Keywords:** Outpatient surgery, Ureteroscopy, Urinary lithiasis

## Abstract

**Background:**

Outpatient surgery is critical to improve health care costs. The aim of the study was to prospectively evaluate the results of outpatient treatment of upper tract urinary stones by rigid or flexible ureteroscopy in a routine care setting.

**Methods:**

A database was created at the creation of the outpatient surgery department. 87 patients underwent 100 ureteroscopic procedures for urinary lithiasis from June 2013 to March 2015.

**Results:**

Most of our patients were male with 53 men (sex ratio M/F 1.13), with a mean age of 52.9 ± 15 years old (23.4–82.4). 44 % of ureteroscopies performed were flexible ureteroscopies, 31 % rigid ureteroscopies and 25 % associated rigid and flexible ureteroscopies. The average stone load was 10.1 ± 5.7 mm (2–30) The mean operating time was 58.3 ± 21.1 min (20–150). 82.9 % of patients had a single urinary stone and 17.1 % (*n* = 14) had 2 or more. 114 stones were treated, 57,1 % intrarenal. There were 6 (6 %) postoperative complications: three Clavien stage 2 infections; three Clavien stage 3b complications (two renal colics requiring ureteral stenting 48 h after discharge and 1 symptomatic perirenal urinoma 48 h after discharge). There was one intraoperative complication (1 %): a ureteral wound with contrast leakage. The rate of transfer to conventional hospitalization was 2.2 %. Stone size influenced the stone-free status (*p* < 0.0001) and the need for more than one session. There was a significant correlation between operative time and stone size above 10 mm (*p* < 0.0001).

**Conclusions:**

Flexible and rigid ureteroscopy are safe and efficient procedures for upper urinary tract stones and can be carried out in an outpatient department. Stone size had an impact on postoperative stone-free status and operative time.

## Background

Ambulatory surgery is performed with admission and discharge of the patient on the same day, with a hospital stay of less than 12 h (no overnight stay). This management is much used in France, which is still lagging behind other countries such as the United States. Ambulatory surgery activity increased by 21 % between 2007 and 2010 in France [1, 2]. Urinary stone disease is common, affecting 3 men to 1 woman, with a peak incidence at between 40 and 50 years of age. Ureteroscopy can handle urinary stones of the ureter as well as in the kidney, being thus a serious alternative to percutaneous nephrolithotomy [3]. Endoscopic treatment of stones is a procedure with low morbidity, between 5 and 10 % [4] and thus it is a procedure performed increasingly in ambulatory surgery in many institutions with patients meeting the outpatient surgery criteria. Data for flexible ureteroscopy (FURS) in ambulatory surgery are few in the literature. Only one study described results of 33 FURS in day-case surgery [5]. FURS is indicated in the treatment of renal kidney stones below 2 cm, in overweight patients, patients with anticoagulants or antiplatelet therapy, urinary stone density greater than 1000 UH, cystine stones, inferior calyx position or if the patient has a particular kidney anatomy such as horseshoe kidney. Rigid ureteroscopy (RURS) is indicated in the treatment of ureteral stones, especially if greater than 1 cm. It can be performed on an emergency basis outside of an infectious episode. [2] The objective of this study was to show that all types of ureteroscopy are an efficient and safe procedure in outpatient surgery.

## Methods

### Design of the study

We collected prospectively the results of rigid and flexible ureteroscopy procedures for urinary stones performed in ambulatory surgery at our University Hospital from June 2013 to February 2015. Ureteroscopy in ambulatory surgery had already been performed as an outpatient modality for several years in our center. At our center, extracorporeal shock wave lithotripsy (ESWL) had not been available for many years. A prospectively maintained database was created at the same time as the opening of our dedicated outpatient surgery department in June 2013. In all there were 103 procedures performed during the study period. The aim of the study was to analyze every day clinical practice. The primary objective was to study the morbidity and early and late conversion rate to conventional hospitalization. The secondary objective was to investigate the efficacy of endoscopic procedures based on the stone-free status assessed by the surgical report or postoperative radiological exam (Ultrasonography or CT-scan) screening for stones under 3 mm in the upper urinary tract. Patients were seen at a postoperative control consultation between 1 and 3 months. Three patients were lost to follow-up because they completed it in another region. We collected demographic data and patient history (ASA score, age, sex, BMI). We also specified the type of ureteroscopy, and characteristics of stones (density, location, size). We noted operative time and the short-term complications.

### Outpatient surgery criteria

All patients met the criteria for outpatient surgery under the SFAR (French Anesthesiology and Reanimation Society) and AFU (French Urology Association) [2] recommendations:

- Patient consent
- Must be escorted home by a third party and should not be alone the first night following surgery
- Must be able to understand and respect the postoperative surveillance guidelines
- Must not have psychiatric problems preventing collaboration with the Ambulatory Surgery Unit
- Must live in acceptable conditions with access to a phone

Patients were initially evaluated by anesthesiologists who validated the procedure feasibility as an outpatient modality. In the same time, 220 ureteroscopies (rigid and flexible) were performed in a conventional hospitalization setting because patients did not meet the criteria for outpatient surgery (see above).

### Surgical technique

For patients with no previous double pigtail stent, surgery was scheduled to attempt a ureteroscopy. If the non-prepared ureter did not allow performing ureteroscopy, a double pigtail stent was placed and the ureteroscopy was scheduled two weeks later. Before surgery, all patients had a sterile urine culture. During the procedure, performed under general anesthesia, all patients received intravenous antibioprophylaxy with 2nd or 3rd generation cephalosporins. Ureteroscopy used an Olympus flexible ureterscope P5 8.4 French (Fr) and an Olympus 7.8 semi-rigid ureteroscope and were performed using a standard safety wire. The majority of stones were treated with a Holmium:YAG laser (Stonelight; AMS) or removed intact with an endoscopic basket. A double pigtail stent was placed as indicated by the operator following a long procedure, in case of repeated endo ureteral maneuvers or in placing the access sheath. After the procedure the patient was discharged after a decision taken in common by the surgeon and the anesthesiologist, at least 4 to 6 h after the ureteroscopy. Postoperative analgesia was ensured by intravenous paracetamol (1 g/6 h) completed by intravenous tramadol 100 mg/8 h) if necessary. This treatment was continued per os after discharge. Criteria to consider hospitalization after surgery (except anaesthetic issues) were an acute urine retention, pain with need for continued intravenous treatment or fever. The double pigtail stent was removed in the outpatient clinic.

### Legislation and statistics

Data collection followed French legislation concerning prospective interventional studies to evaluate routine care (Article Art. L1121-1-2 of French Public Health Code (Code de santé publique français)). Institutional review board approval was obtained to prospectively collect data on patients who underwent Ureteroscopy. The study did not require submitting to a Consultative Committee for Persons’ Protection in Biomedical Research (CCPPRB). Patients were informed verbally and received an information document edited by the French Urology Association (http://urofrance.org/nc/lurologie-grandpublic/fiches-patient/resultats-de-la-recherche/html/ureteroscopie.html). Categorical variables were analyzed using the chi-square test and Fisher’s exact test when applicable. Continuous variables were analyzed parametrically using Student’s t-test and non-parametrically using the Kruskal–Wallis test or the Mann and Whitney test. For univariate analysis, *p* < 0.05 defined statistical significance.

## Results

In total, 100 procedures in 87 patients were included. The patients all meet the criteria for outpatient surgery; their characteristics are summarized in Table 1. The majority of patients were male with 53 men (sex ratio M/F 1.13), with a median age of 56 years old (Interquartile range (IQR) 39–64.5; min 23.4-max 82.5). Patients were exclusively ASA 1 (*n* = 62; 71.3 %) and ASA 2 (*n* = 25; 28.7 %).

**Table 1:** Patients characteristics by treatment groups

		Rigid ureteroscopy	Flexible ureteroscopy	Rigid + flexible ureteroscopy	*p*
		*N* = 31	*N* = 44	*N* = 25	
Multiple procedures		1	9	3	0.05
Gender	F	8 (26.7 %)	17 (44.7 %)	9 (40.9 %)	
	M	22 (73.3 %)	18 (55.3 %)	13 (59.1 %)	
Median age at TT (years old)		54.2 (35.8–66.2) (25.3–82.4)	43.4 (25.1–60) (21–76.4)	54.7 (39–61) (26.8–76.4)	0.07
ASA 1		22	30	10	
ASA 2		8	5	12	
Median BMI (kg/m^2^) (IQR) (Min-max)		25.4 (24–28.3) (17.3–37.8)	26 (24.3–29.3) (18.9–38.1)	26.8 (23.9–28) (21–39.9)	1
History of urinary stones treatment (same side)	ESWL	1	4	1	0.014
	URS	2	4	0	
	PCNL	0	3	0	
Preoperative JJ stent		24 (77.4 %)	35 (79.5 %)	23 (92 %)	0.2
Median time between diagnosis and TT (days) (IQR) (min-max)		58 (30–92.5) (7–146)	63 (33–80.5) (14–127)	57.4 (41–80) (14–213)	0.9
Stones size (mm) (IQR) (min-max)		7 (6–10) (4–30)	12.5 (10–17.5) (6–30)	8 (6.5–14) (4–34)	0.0008
Median stones density (UH) (IQR) (min-max)		1000 (900–1200) (550–1330)	920 (800–1000) (500–1580)	895 (525–1150) (433–1450)	0.13
Median operative time (min) (IQR) (min-max)		41.5 (31–59.5) (10–90)	60 (52.5–79) (20–150)	60 (46.2–66.7) (31–90)	<0.0001
Postoperative double pigtail stent		19 (61.3 %)	33 (75 %)	14 (56 %)	0.2
Stone free		27/30 (90 %)	25/35 (71.4 %)	21/22 (95.4 %)	0.03
Postoperative complications	Clavien 2	0	2 APN	0	0.3
			1 APr		
	Clavien 3	2 Renal colics	1 Urinoma	0	

### Descriptive analysis of operating characteristics

The median time between the date of diagnosis and ureteroscopy was 82.5 days (IQR 57.2–119.3; min 13-max 739). 44 % of ureteroscopies were FURS, 31 % were RURS and 25 % associated rigid and flexible ureteroscopy if there were several locations or the original ureteral stone was flushed in the kidney (22 patients). One failure of FURS (procedure stopped after 30 min) was secondary to ureteral wound probably during the ascension of the access sheath. The median operating time was 60 min (IQR 45–75; min 20- max 150). A majority of patients had a double pigtail stent preoperatively placed during an acute episode of renal colic (*n* = 52) or obstructive pyelonephritis (*n* = 14) or simply to prepare the endoscopic procedure (*n* = 22). Twelve procedures could be performed without a previous double pigtail stent. The median time between double pigtail stent placement and ureteroscopy was 63.5 days (IQR 35–92; min 7- max 213). Postoperatively a double pigtail stent was left in place after 66 procedures (66 %).

### Urinary stone characteristics

**Fig. 1:**
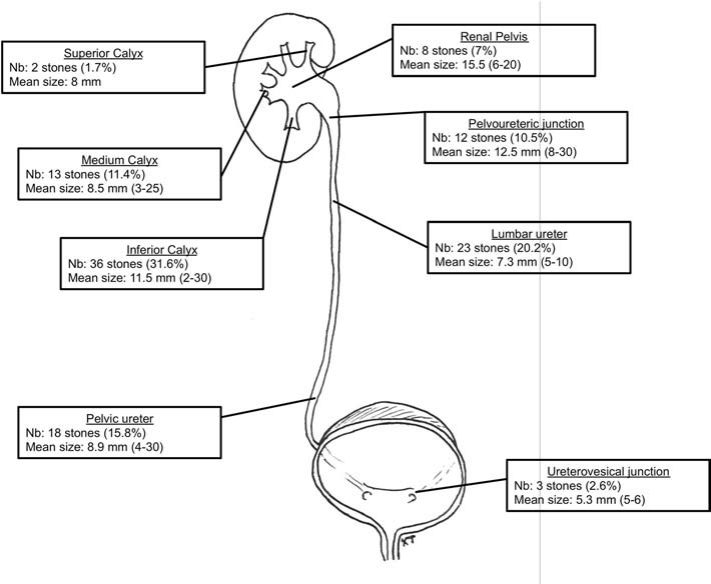
Stone size and stone location

82.9 % of patients were carrying a single stone and 17.1 % (*n* = 14) two or more. One hundred fourteen stones were treated (Fig. 1). The distribution and mean size of urinary stones are presented in Table 2. The mean stone density evaluated on preoperative CT scans was 964.9 ± 286.7 HU. The median stone load was 8 mm (IQR 6,3–13; min 2- max 30). Kidney urinary stones under 6 mm in size were processed at the same time as the main stone.

**Table 2:** Patients characteristics by stone localization

		Distal ureter stones	Proximal ureter stones	Renal stones	*p*
		*N* = 16	*N* = 22	*N* = 62	
Multiple procedures		0	2	11	
Rigid ureteroscopy		14	12	3	
Flexible ureteroscopy		0	2	43	
Both		2	8	16	
Gender	Female	6	8	24	
	Male	10	12	27	
Median age at TT (years old) (IQR) (Min-max)		54 (35–54) (25–82)	52 (23–67) (23–80)	57 (25–65) (25–76)	0.8
ASA 1		14	18	34	
ASA 2		2	2	17	
Median BMI (kg/T^2^) (IQR) (Min-Max)		25 (24–28) (21–32)	27 (25–29) (17–40)	26 (24–28) (19–38)	0.47
History of urinary stones treatment (same side)	ESWL	0	0	6	0.035
	URS	1	0	5	
	PCNL	0	0	3	
Preoperative JJ stent		14 (87.5 %)	18 (81.8 %)	49 (79 %)	0.74
Median time between diagnosis and TT (days) (IQR) (Min-max)		86 (50–109) (13–289)	67 (38–93) (21–151)	93 (65–156) (25–739)	0.053
Median Stone size (mm) (IQR) (Min-max)		7 (5.3–10) (4–30)	7 (6.3–8) (4–10)	10 (6.6–13) (2–30)	0.014
Median stone density (UH) (IQR) (Min-max)		1003 (930–1125) (550–1310)	1000 (660–1200) (433–1330)	943 (618–1000) 500–1580)	0.24
Median operative time (min) (IQR) (Min-max)		40 (30–45) (20–60)	45 (35–60) (10–90)	60 (50–75) (20–150)	<0.0001
Postoperative double pigtail stent		8 (50 %)	15 (68.2 %)	43 (69.35 %)	0.33
Stone free after multiple procedures		15/16 (93.7 %)	19/20 (95 %)	38/51 (74.5 %)	0.053
Postoperative complications	Clavien 2		1 AProst	2 APN	
			1 Renal colic	1 Renal colic	
	Clavien 3			1 Urinoma	

### Postoperative complications

Postoperative complications were first of all infectious (Clavien stage 2): acute prostatitis at day 13, 2 pyelonephritis 48 h after surgery. Three complications were Clavien stage 3b: 2 renal colic requiring ureteral stenting 48 h after ambulatory discharge, and a symptomatic perirenal urinoma 48 h after surgery. Complications rate was 6 %.

There was one intraoperative complication (1 %): an ureteral lesion with contrast leakage requiring ureteral stent and the cessation of the procedure after 30 min.

The conventional hospitalization conversion rate was 2 % (2/100) and included the patient with an intraoperative ureteral wound and a patient with poorly controlled postoperative pain. Gvien the low rate of complications it was not possible to identify patients or procedures at risk of complications.

### Statistical analysis and follow up

The mean operative time of patients who had postoperative complications or who were converted to conventional hospitalization was not different from the mean operative time of other patients (64,6 vs. 57,2 min; *p* = 0.4). In the same way, ureteroscopy type (rigid, flexible or both) was not associated to postoperative complications (*p* = 0.3). Median follow up was 3.5 months (IQR 2.1-5.5; min 0.7- max 9.4). Seventy-two patients were assessed post operatively for residual fragments by CT-scan and 15 by US. On the one hand, studying the patient groups “stone Free” and the patient group “not stone free,” we found no significant difference in the number of urinary stones before surgery (*p* = 0.37) nor in terms of their density (*p* = 0.85). On another hand, the impact of stone size on the stone-free status and the need for one or more sessions was highly significant (*p* < 0.0001). In addition, there was a significant correlation between operative time and stone size (*p* < 0.0001; *r* = 0.59), but not between the operating time and stone density (*p* = 0.8). Bigger urinary stones were treated by FURS (*p* = 0.0008) on patients with longer histories of urinary stone treatment (*p* = 0.014). Mean operative time was higher for FURS (*p* < 0.0001). At the end of 34 procedures, double pigtail stent was not required. Median time of ureteral catheter removal after ureteroscopy was 22 days (IQR 17.5-34.5; min 8- max 119).

## Discussion

In Urology, several procedures can be done in outpatient surgery: female urethral sling, ACT balloons, male genital organ surgery, and prostate laser surgery. Radical prostatectomies and nephrectomies are currently under investigation [6, 7] as well as Percutaneous Nephrolithotomy [8, 9] with case reports and small series. Ambulatory surgery is encouraged in all countries by different national health systems with a view to its economic advantages [2]. An american study evaluated that ambulatory surgery could generate savings of 363 to $1000 US dollars per outpatient case [10]. Rigid or semi-rigid ureteroscopy are routinely performed in many countries especially in the US. Indeed in 1994, Wills asked whether ureteroscopy could be performed as an outpatient surgery [11]. Most of the studies of ureteroscopy describe procedures during conventional inpatient hospitalization or a day-case procedure with an overnight stay. In a strictly defined outpatient setting, patient admission and discharge should occur on the same day. The first study describing results of ambulatory rigid ureteroscopy with laser stone fragmentation was published in 1998 by Yip [12]. The complication rate in 69 patients was 10 % with 6 % of unplanned readmission. This study demonstrated that rigid ureteroscopy for ureteric stones can be performed in an outpatient surgery department, with an acceptable rate of unplanned readmissions and an acceptable rate of complications. Several other studies followed confirming these results [13, 14]. Others publications studied factors influencing JJ stent placement after outpatient procedures [15], or preoperative predictors of postoperative events [16]. Ureteroscopy had to be compared to other outpatient procedures to treat upper urinary tract stones, namely extracorporeal shockwave lithotripsy [17, 18]. Jeong reported that shockwave lithotripsy was more painful with a lower rate of stone free patients.

Ureteroscopy is not dependent on stone radiolucent characteristic: any kind of urinary stone can be treated. FURS is a widely used procedure, but documentation on the safety and efficacy of this treatment modality in an outpatient setting is scarce. There were only two studies in the literature analyzing results of FURS in a true outpatient setting, but these studies were retrospective reviews. Bromwich [5] reported a study with the same design than ours. 64 rigid and flexible ureteroscopies were performed in outpatient surgery. 45 patients were treated for urinary stones including 13 FURS. Rate of immediate admission was 6.25 % (*n* = 4) mostly following postoperative uncontrolled pain. Rate of unplanned readmission after discharge was 4.7 % (*n* = 3) with one urinary retention, one clot retention and one for social reasons. Tan [19] studied the rate of immediate unplanned hospital admission and factors associated with admission for outpatient ureteroscopy for stone disease. For 1798 consecutive outpatient ureteroscopic procedures for urolithiasis, there were 70 immediate unplanned admissions (3.9 %). The authors found after multivariate analysis that the significant factors associated with unplanned admission were: previous admission related to stones, history of psychiatric illness and bilateral procedure. Unfortunately, the number of FURS included in this study was not specified. In the latest study of outpatient ureteroscopy for kidney stones, 82 % of the patients were considered stone free after one procedure and an overall stone-free rate of 85 % was found. Several studies have found stone-free rate higher than 90 % after one procedure [5, 19, 20]. However, most of these studies included small study populations and the outpatient setting was unclear, which makes comparison of the findings difficult. Moreover there is, no standardized definition of “stone-free rate” in the literature, and this fact makes comparisons across studies difficult [21]. At the time of surgery, 51.7 % of the patients had renal stones, which fact could explain variability in operative time (20 to 150 min). This is well known, as many steps in this procedure, such as ascension of the access sheath, laser stone vaporization and basket removal of stone fragments can take varying times and are difficult to predict. This high variability in operating times shows that ureteroscopic stone removal is a challenging procedure with a tight schedule in an outpatient surgery department. Accurately estimating operating times is essential in an outpatient surgery department. Recently, Lasselin [22] demonstrated that rate of ureteroscope damage is associated with operative time and cumulative duration of intervention. Its therefore important to limit ureteroscopy operating time in outpatient surgery in order to limit ureteroscope damage and to ensure good patient outcomes. With a low rate of surgical and anesthesiology complications, these procedures can be repeated to achieve upper urinary tract stone treatment whatever the initial stone load. The remaining question is the rate of ureteral stricture which is a medium and long follow up complication after ureteroscopy, occurs in 3–6 % of the surgeries and could be treated conservatively with a JJ stent according to Preminger [23]. The major strength of the present study is the availability of detailed medical records for each patient, and the prospective design that provided very comprehensive data. There are some limitations to this study. Only complications leading to contact with the hospital were registered and patients could have experienced other complications at home. Another limitation is the lack of a standardized definition of stone-free status.

## Conclusion

Rigid as well as flexible ureteroscopy is a safe and efficient procedure that can easily be carried out in an outpatient department to treat upper urinary tract stones. It can reduce the need for admissions and thus cut healthcare costs. Most kidney urinary stones can be treated by flexible ureteroscopy in outpatient surgery with a low rate of complications and no difference from rigid ureteroscopy. Multiple locations and high stone load were associated with multiple procedure with no increase in morbidity rate. If ESWL is not available, FURS is a good treatment in an outpatient setting whatever the stone size and location.

### Ethics approval and consent to participate

Study approved by Caen University Hospital ethic committee (no reference number provided).

Patients were informed verbally and received an information document edited by the French Urology Association.

## Abbreviations

FURS: flexible ureteroscopy
ESWL: extracorporeal shock wave lithotripsy
RURS: rigid ureteroscopy
